# Atypical parathyroid adenoma: A case report

**DOI:** 10.1002/ccr3.6785

**Published:** 2022-12-22

**Authors:** Chayma Besrour, Imen Rojbi, Nadia Mchirgui, Ibtissem Ben Nacef, Karima Khiari

**Affiliations:** ^1^ Department of Endocrinology Charles Nicolle Hospital Tunis Tunisia; ^2^ Faculty of Medicine of Tunis Tunis El Manar University Tunis Tunisia

**Keywords:** atypical parathyroid adenoma, case report, hypercalcemia, hyperparathyroidism

## Abstract

The atypical parathyroid adenoma is a histological diagnosis. It is a parathyroid tumor with atypical histological features different from an adenoma and not similar enough to be considered as a carcinoma. It has an uncertain malignant potential. We report the case of a 55‐year‐old woman, diagnosed with severe hypercalcemia during her follow‐up with the rheumatologist for osteoporosis. The laboratory testing led to the detection of a very important level of parathormone PTH 1325.62 pg/ml (20 fold the normal level), confirming the diagnosis of primary hyperparathyroidism. Investigations of the potential causes led to the presence of a large left inferior parathyroid adenoma. The patient was operated and on the examination of the removed tissue, the histopathological examination concluded to an atypical parathyroid adenoma, and the post‐operatory PTH level dropped significantly to 152 pg/ml. The atypical parathyroid adenoma is a very rare tumor, and the diagnosis is still a challenge, the outcome of patients is not well known yet, there for the surveillance is important and must be regularly.

## Graphical abstract

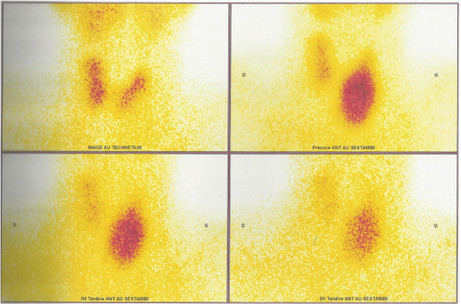

## 1 INTRODUCTION

Primary Hyperparathyroidism is one of the most frequent causes of hypercalcemia. It is an endocrine disorder, most often due to the presence of a parathyroid adenoma (80%–85%), sometimes to parathyroid hyperplasia (10%–15%) and less frequently to atypical parathyroid adenoma (APA) and parathyroid carcinoma (1.2%–1.3% and 1% respectively).^1^ Although the true incidence of APA is unknown. APA is a parathyroid tumor with atypical histological features different from an adenoma and not similar enough to be considered as a carcinoma.^1^ It has an uncertain malignant potential.

## 2 PATIENT AND OBSERVATION

### 2.1 Patient information

We report the case of a 55‐year‐old woman descended from a first degree consanguineous marriage, with a primary ovarian insufficiency at the age of 38 years old (FSH 57.7 mUI/L [3–9 mUI/L], LH 22.71 mUI/L [2–10 mUI/L]) treated by her rheumatologist for osteoporosis with vitamin D supplementation and bisphosphonate treatment. She was referred to us after discovering a severe hypercalcemia (Ca 3.68 mmol/L [2.2–2.6 mmol/L]). She was immediately hospitalized in our department.

### 2.2 Clinical findings

The patient was dehydrated but with no signs of confusion. The cardiac assessment was normal; the blood pressure was at 120/80 mmHg, the electrocardiogram did not show an abnormal cardiac rhythm (70 beats per minute, normal QT interval).

### 2.3 Diagnostic approach

The biology confirmed the high level of calcium at 3.43 mmol/L, associated to a low level of phosphorus at 0.53 mmol/L [0.74–1.50 mmol/L], a low level of 25‐hydroxyvitamin D at 12.5 ng/ml [30‐50 ng/ml], a normal albumin level at 43 g/L, and a very important level of parathormone PTH 1325.62 pg/ml [10‐55 pg/ml] (20 fold the normal level), confirming the diagnosis of primary hyperparathyroidism. The renal assessment was also normal (creatinine level at 62 μmol/L, no nephrolithiasis), except for an hypercalciuria at 6.44 mmol/L/24 h (>5.7 mmol/L/24 h, weight 57 kg).

**FIGURE 1:**
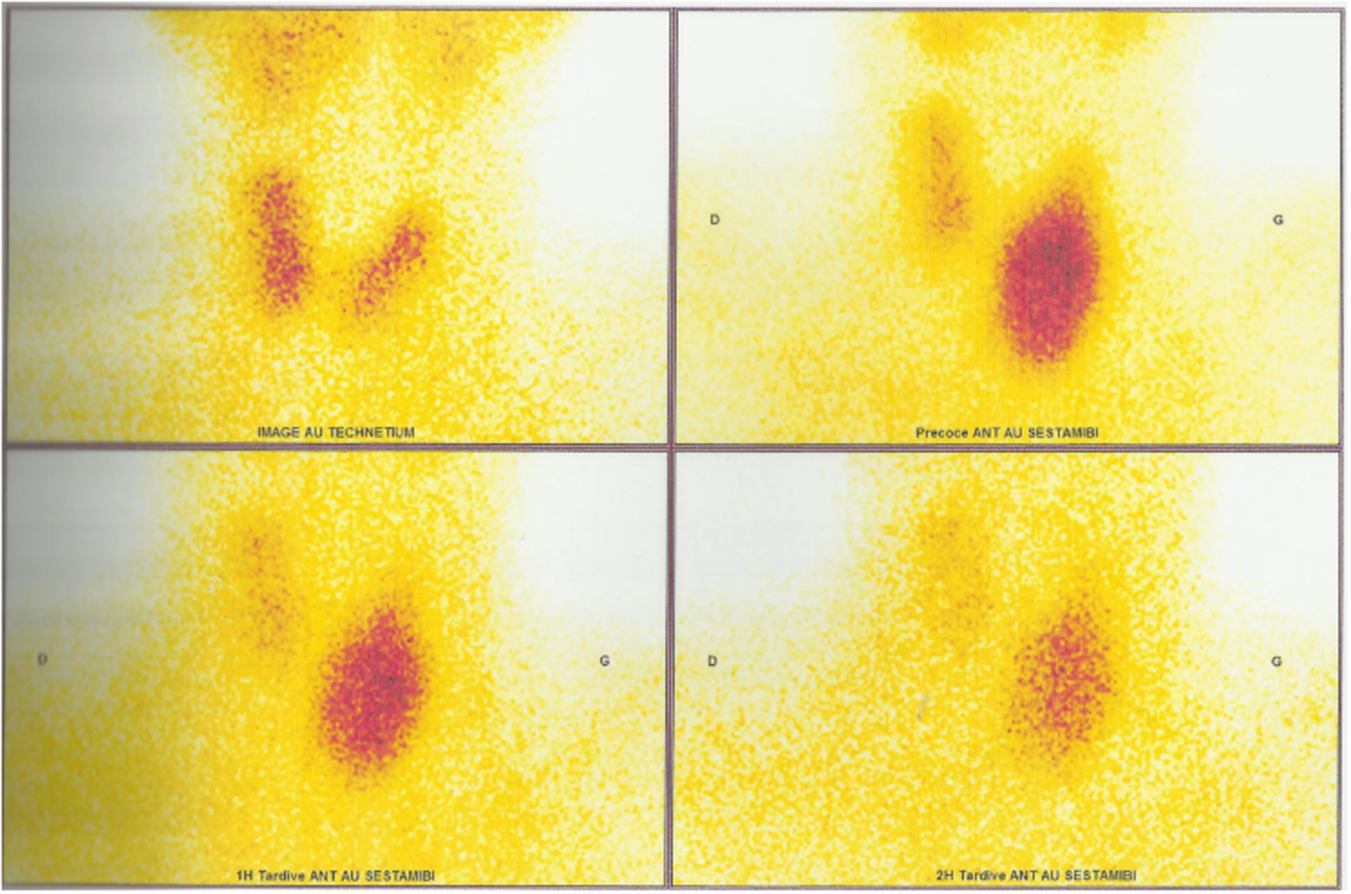
Parathyroid scan sestamibi

The cervical ultrasound revealed a left hyperechoic, heterogeneous parathyroid mass, measuring 2.05 × 3.97 × 3.18 cm in diameter with a normal thyroid gland, and the Technetium 99 SESTAMIBI‐Scintigraphy showed a large left inferior parathyroid adenoma (Figure 1).

In front of this large mass, the severe hypercalcemia and the very high level of PTH, the malignancy was suspected, and we completed the exploration by dosing two biomarkers of malignancy usually found in the parathyroid carcinoma, the alkaline phosphatase (ALP) which was at 420 UI/L [44–147 UI/L] (3 fold normal level) and the βHCG was in the upper limit of the normal range at 4.09 mUI/ml (<5 mUI/ml).

### 2.4 Therapeutic intervention

Before referring the patient to surgery, she received an intravenous bipohosphonate therapy (Zoledronic acid) to control the hypercalcemia, and vitamin supplementation to prevent the hungry bone syndrome, then she underwent a surgery under general anesthesia with kocher's incision. The surgeons found a voluminous lower left parathyroid nodule very adhering to the thyroid gland, muscles and esophagus. They did a parathyroidectomy with left monobloc loboisthmectomy.

The extemporaneous examination was in favor of a parathyroid adenoma and healthy thyroid lobe.

On the examination of the removed tissue, we received a loboisthmectomy piece measuring 3 × 2 × 0.5 cm and joined and occupied almost entirely by a parathyroid nodule of 5 × 3.2 × 2.5 cm with poorly defined and friable contours. Microscopic examination shows proliferation of small monomorphic cells, non‐atypical and non‐mitotic associated with a low contingent of eosinophilic cells. Cells are organized into tubular structures within a richly vascularized stroma. This proliferation is fragmented by wide bands of fibrosis associated with hemosiderin deposits. Focally, this proliferation is poorly limited adherent to the thyroid parenchyma and to the adipose tissue and the striated muscle. Absence of vascular embolism and tumor necrosis.

These histological aspects are consistent with an atypical parathyroid adenoma of 5 × 3.2 × 2.5 cm.

### 2.5 Evolution

After the surgery, there were no postoperative complications, and the level of calcium normalized to 2.25 mmol/L and the PTH level dropped significantly to 152 pg/ml. The patient consulted 2 months after, she was in good health and the biology showed a calcium level at 2.2 mmol/L, Phosphorus level at 1.19 mmol/L, ALP level at 220 UI/L, and PTH level at 100 pg/ml.

## 3 DISCUSSION

Parathyroid tumors are very rare and affect between 0.1% and 0.3% of the general population.^2^ Among them APA is exceptional and is responsible for 1.2%–1.3% of primary hyperparathyroidism (PHPT),^1^ it has an unknown malignant potential.

It predominates in the female gender with a sex ratio 1.5:1. It usually occurs in the fourth decade (median age 44 years), one decade earlier than the benign parathyroid disease.^2^

The pathogenic mechanisms responsible for the APA are still unknown and only few studies tried to explain.

The clinical profile is similar to that found in severe hypercalcemia. The symptoms include fatigue, asthenia, anxiety, neurocognitive decay, anorexia, constipation, nausea and vomiting, bone pain, nephrolithiasis, hypertension, and sometimes depression.^3^

Some signs are not habitual in benign hyperparathyroidism and there for should be taken seriously, such as dysphonia and dysphagia which are signs of local compression, also a palpable neck mass are not rare to find (15% on physical examination), usually they are more than 2.5 cm.^2^

More over the biology shows a severe hypercalcemia with a markedly elevated PTH level. Alkaline phosphatase (ALP) activity can be also superior to upper normal range, and the human chorionic gonadotropin (HCG) plasma level may also be elevated (normal range non‐pregnant female or male <4 IU/L).^4^ This profile is more similar to parathyroid carcinoma than to a typical benign adenoma which should attract attention to the possible malignant nature of the mass.

As for the radiological exploration, neck ultrasound is the first line imaging, describing the mass; nature cystic or solid, measures and its anatomical contact with adjacent structures. Associated with Single‐photon emission computed tomography (SPECT/CT), they represent the recommended radiological examination for the localization the tumor pre‐operatively.^5^, ^6^, ^7^

As for the management of the tumor, surgery is the only treatment and it consists to a total resection of the lesion “en bloc resection.” Then, it is the role of the histopathological examination to confirm the diagnosis of the APA and to differentiate it from other differential diagnoses such as a benign typical parathyroid adenoma and the parathyroid carcinoma. According to the world health organization, the APA is defined as a parathyroid tumor that do not show locally advanced growth or metastases, but may show cell atypia, fibrotic tissue, trabecular growth, fibrotic capsular involvement, and increased mitotic rate.^8^ Immunohistochemistry and molecular biology are interesting in this case and can bring additional help to the diagnosis.

Sometimes, despite the histopathological examination of the tumor, the differentiation between an APA and a PC may remain difficult and only the surveillance can define the diagnosis. PC has shown a recurrence rate >50% that most occur 2–3 years after the surgery.^9^ As for the APA, many series evaluated the long‐term outcome and did not show any recurrence over a mean follow‐up of 5 years.^10^ But no specific guidelines for the surveillance rhythm after parathyroid surgery exist so far.

## 4 CONCLUSION

The atypical parathyroid adenoma is a very rare tumor that shares similarities with both typical adenoma and parathyroid carcinoma making the diagnosis challenging. Moreover, the outcome of patients is not well known yet; therefore, it must be considered as“a tumor of uncertain malignant potential” justifying a regularly careful and strict follow‐up.

## AUTHOR CONTRIBUTIONS

Chayma Besrour contributed to the bibliographic research and to the writing of the article. Imen Rojbi contributed to the layout of the references. Nadia Mchirgui participated in the care of the patient and the correction of the article. Karima Khiari and Ibtissem Ben Nacef participated in the correction of the article. All authors have read and approved the final version of the manuscript.

## FUNDING INFORMATION

None.

## CONFLICT OF INTEREST

The authors declare no competing interest.

### CONSENT

Written informed consent was obtained from the patient for the publication for this case report and the accompanying image.

REFERENCES1

Galani
A
, 
Morandi
R
, 
Dimko
M
, et al. Atypical parathyroid adenoma: clinical and anatomical pathologic features. World J Surg Oncol. 2021;19:19. doi:10.1186/s12957-021-02123-7
33472651PMC78187512

Cetani
F
, 
Marcocci
C
, 
Torregrossa
L
, 
Pardi
E
. Atypical parathyroid adenomas: challenging lesions in the differential diagnosis of endocrine tumors. Endocr Relat Cancer. 2019;26:R441‐R464. doi:10.1530/ERC-19-0135
310857703
SFEndocrino. Accessed March 1, 2021. http://www.sfendocrino.org/article/832/poly2016‐item‐266‐ndash‐ue‐8‐hypercalcemie
4
hCG (Human Chorionic Gonadotrophin)/Blood Sciences Test/Exeter Clinical Laboratory International. Accessed March 7, 2021. https://www.exeterlaboratory.com/test/hcg‐human‐chorionic‐gonadotrophin/
5

Saponaro
F
, 
Cetani
F
, 
Repaci
A
, et al. Clinical presentation and management of patients with primary hyperparathyroidism in Italy. J Endocrinol Invest. 2018;41:1339‐1348. doi:10.1007/s40618-018-0879-z
296164196

Wilhelm
SM
, 
Wang
TS
, 
Ruan
DT
, et al. The American Association of Endocrine Surgeons Guidelines for definitive Management of Primary Hyperparathyroidism. JAMA Surg. 2016;151:959‐968. doi:10.1001/jamasurg.2016.2310
275323687

Lavely
WC
, 
Goetze
S
, 
Friedman
KP
, et al. Comparison of SPECT/CT, SPECT, and planar imaging with single‐ and dual‐phase (99m)Tc‐sestamibi parathyroid scintigraphy. J Nucl Med. 2007;48:1084‐1089. doi:10.2967/jnumed.107.040428
175749838

DeLellis
RA
, 
Lloyd
RV
, 
Heitz
PU
, 
Eng
C
. Pathology and Genetics of Tumours of Endocrine Organs. IARC Press; 2004.9

Marcocci
C
, 
Cetani
F
, 
Rubin
MR
, 
Silverberg
SJ
, 
Pinchera
A
, 
Bilezikian
JP
. Parathyroid carcinoma. J Bone Miner Res. 2008;23:1869‐1880. doi:10.1359/jbmr.081018
19016595PMC327634410

McCoy
KL
, 
Seethala
RR
, 
Armstrong
MJ
, et al. The clinical importance of parathyroid atypia: is long‐term surveillance necessary?
Surgery. 2015;158:929‐935; discussion 935–936. doi:10.1016/j.surg.2015.06.022
26210223

## Data Availability

None.
